# High multiple mutations of *Plasmodium falciparum*-resistant genotypes to sulphadoxine-pyrimethamine in Lagos, Nigeria

**DOI:** 10.1186/s40249-020-00712-4

**Published:** 2020-07-11

**Authors:** Hong Quan, Uche Igbasi, Wellington Oyibo, Sunday Omilabu, Shen-Bo Chen, Hai-Mo Shen, Chukwuma Okolie, Jun-Hu Chen, Xiao-Nong Zhou

**Affiliations:** 1 National Institute of Parasitic Diseases, Chinese Center for Disease Control and Prevention, Chinese Center for Tropical Diseases Research, WHO Collaborating Center for Tropical Diseases, National Centre for International Research on Tropical Diseases, Ministry of Science and Technology, Key Laboratory of Parasite and Vector Biology, Ministry of Health, Shanghai, 200025 People’s Republic of China; 2 National Institute of Parasitic Diseases, Chinese Center for Disease Control and Prevention⁃Shenzhen Center for Disease Control and Prevention Joint Laboratory for Imported Tropical Disease Control, Shanghai, 200025 People’s Republic of China; 3 Center for Infectious Diseases Research, Microbiology Department, Nigerian Institute of Medical Research, 6 Edmund Crescent, Yaba, Lagos, Nigeria; 4 ANDI Center of Excellence for Malaria Diagnosis, Department of Medical Microbiology and Parasitology, College of Medicine, University of Lagos, Lagos, Nigeria; 5 Department of Medical Microbiology and Parasitology, College of Medicine, University of Lagos, Lagos, Nigeria; 6 Department of Surveying and Geoinformatics, Faculty of Engineering, University of Lagos, Lagos, Nigeria

**Keywords:** *Plasmodium falciparum*, Antimalarial drug resistance, Sulphadoxine–pyrimethamine, Dihydrofolate reductase, Dihydropteroate synthase

## Abstract

**Background:**

*Plasmodium falciparum*-resistance to sulphadoxine-pyrimethamine (SP) has been largely reported among pregnant women. However, the profile of resistance markers to SP dihydrofolate reductase (*dhfr*) and dihydropteroate synthase (*dhps*) in the general population are varied and not frequently monitored. Currently, SP is used as partner drug for artemisinin combination therapy (SP-artesunate) in some sub-Saharan African countries or as a prophylactic drug in intermittent preventive treatment of malaria during pregnancy and infants and in seasonal malaria chemoprevention (SMC). Profiling of *P. falciparum*-resistant genotypes to SP is dynamic and critical in providing data that would be useful for malaria control programmes. This study assessed the profile of *dhfr* and *dhps* genes genotypes among individuals with malaria in Lagos, Nigeria.

**Methods:**

Molecular markers of SP resistance were identified by nested PCR and sequenced among malaria positive dried blood spots (DBS) that were collected from individuals attending health facilities from January 2013 to February 2014 and during community surveys from October 2010 to September 2011 across different Local Government Areas of Lagos State, Nigeria.

**Results:**

A total of 242 and 167 samples were sequenced for *dhfr* and *dhps*, respectively. Sequence analysis of *dhfr* showed that 95.5% (231/242), 96.3% (233/242) and 96.7% (234/242) of the samples had N51I, C59R and S108N mutant alleles, respectively. The prevalence of *dhps* mutation at codons A437G, A613S, S436A, A581G, I431V and K540E were 95.8% (160/167), 41.9% (70/167), 41.3% (69/167), 31.1% (52/167), 25.1% (42/167), and 1.2% (2/167) respectively. The prevalence of triple mutations (CIRNI) in *dhfr* was 93.8% and 44.3% for the single *dhps* haplotype mutation (SGKAA). Partial SP-resistance due to quadruple *dhfr-dhps* haplotype mutations (CIRNI-SGKAA) and octuple haplotype mutations (CIRNI-VAGKGS) with rate of 42.6% and 22.0%, respectively has been reported.

**Conclusions:**

There was increased prevalence in *dhfr* triple haplotype mutations when compared with previous reports in the same environment but aligned with high prevalence in other locations in Nigeria and other countries in Africa. Also, high prevalence of *dhfr* and *dhps* mutant alleles occurred in the study areas in Lagos, Nigeria five to eight years after the introduction of artemisinin combination therapy underscores the need for continuous monitoring.

## Background

The efficacy of antimalarial medicines is critical to the implementation of effective malaria case management where patients confirmed to have malaria parasites are treated promptly. Consequently, failing antimalaria medicines due to parasite resistance will greatly affect the attainment of the case management goal. Resistance to antimalarial drugs has been described for *Plasmodium falciparum*, the predominant *Plasmodium* species in Africa [1]. Over a decade, sulphadoxine-pyrimethamine (SP) was the second-line treatment medicine while chloroquine (CQ) served as the first-line antimalarial medicine for the treatment of uncomplicated *P. falciparum* malaria [2]. *P. falciparum*, unfortunately developed resistance to both widely used medicines and are not currently recommended for the treatment of malaria as monotherapies in the general population. The malaria parasite’s resistance to SP is due to point mutations in target enzymes, dihydrofolate reductase (*dhfr*) and dihydropteroate synthase (*dhps*) [3]. Resistance to SP and CQ were reported at different times in the history of anti-malarial medicine resistance [4, 5].

Currently, SP is used as a partner drug for antimalarial drug resistance (ACT, such as SP-artesunate), seasonal malaria chemoprevention (SMC) in areas where it is recommended, intermittent preventive treatment of malaria in infants and children (IPTi & c) in some sub-Saharan African countries, and as intermittent preventive treatment of malaria in pregnancy (IPTp) [6]. The spread of SP resistance may compromise the effectiveness of intermittent preventive treatment of malaria in pregnancy (MiP) with SP (IPTp-SP) and other interventions including SMC across Africa. In West Africa, SP resistant genes of *P. falciparum* have been reported, and IPTp-SP remains the interventional strategy for the prevention of malaria in pregnancy [7]. Studies in Nigeria have reported varying mutant combinations with over 50.0–96.9% prevalence of SP-resistant mutations in the last decade [8–10].

SP acts primarily on the schizonts during the hepatic and erythrocytic phases of the plasmodia life cycle [11], by inhibiting enzymes necessary for parasite folate biosynthesis. Pyrimethamine acts by inhibiting *dhfr* in the parasite [12], thus preventing the biosynthesis of purines and pyrimidines, while sulphadoxine binds the enzyme *dhps* [13], inhibiting the use of para-aminobenzoic acid during the synthesis of dihydropteroic acid. When combined the two key stages in DNA synthesis in the plasmodia are prevented consequently, cell division and reproduction are halted. As these two drugs target the same pathway and act synergistically, they are usually given in combination as SP but referred to as monotherapy [14].

Mutations in the *dhfr* and *dhps* genes of *P. falciparum* parasites have been associated with decreased parasite sensitivity to the anti-folate drugs. In both genes, each successive mutation has been shown to incrementally increase the parasite’s tolerance to the drug in vitro [14]. A change from wild type serine 108 to asparagine108 (S108N) in *dhfr* is sufficient to cause low level pyrimethamine resistance both in vitro and in vivo [15]; this represents the initial and critical mutation for pyrimethamine. Additional mutation(s) at positions 50: C50R, 51: N51I, 59: C59R and I164L synergistically increase the levels of resistance [16, 17].

Furthermore, mutation from alanine to glycine at codon 437 (A437G) for *dhps* represents the critical mutation for sulphadoxine resistance and additional mutation(s) at positions 436 (S436A/F), 540 (K540E), 581 (A581G), and 613 (A613S/T) have been associated with decreased parasite sensitivity to the sulpha drugs including sulphadoxine and dapsone [13, 18, 19]. Mutations at codons 437 and 540 of *dhps* play the most significant role in sulphadoxine resistance among African parasites. In East and South Africa, mutations at the 437 and 540 codons are found together while in West and Central Africa the 437 is found on its own [20]. However, mutation at codon 431 (I431V) has been scarcely reported. It was first reported among imported malaria infections that originated from Nigeria in 2009 [21] and pregnant women from Cameroon in 2015 [22, 23], though its effect on parasite susceptibility to SP is yet to be fully described [10].

It has been demonstrated that the accumulation of single nucleotide polymorphisms (SNPs) in *dhfr* and *dhps* genes increases the levels of SP resistance in vivo [23]. In West and Central Africa, a triple mutant genotype of *dhfr* (N51I, C59R and S108N) combined with the A437G mutation in the *dhps* gene has been related to SP treatment failure [24]. Another significant predictor of SP treatment failure is the quintuple mutant genotype, which includes the *dhfr* triple mutations (N51I, C59R and S108N) combined with the *dhps* double mutations (A437G + K540E) [25–27].

In Nigeria, high prevalence of triple mutant genotype of *dhfr* (N51I, C59R and S108N) combined with A437G mutation in the *dhps* gene have been reported [10], but reports of quintuple *dhfr*/*dhps* mutation comprising of (N51I, C59R and S108N) plus (A437G + K540E) is scarce [10].

SP-resistant parasites could be classified as “partially resistant”, “fully resistant” and “super resistant” [28]. The parasites are classified based on the combination of mutations they carry in the two genes (*dhfr* and *dhps*). The quadruple combination of triple mutation, *dhfr* N51I, C59R, S108N and *dhps* A437G, confers partial resistance; the quintuple combination of triple mutations, *dhfr* N51I, C59R, S108N and double mutation, *dhps* A437G, K540E, confers full resistance; and the sextuple combination of triple mutation, *dhfr* N51I, C59R, S108N and triple mutation, *dhps* A437G, K540E, A581G, confers super resistance [28]. These haplotype mutations affect the outcome of IPTp and IPTi [28] .^.^

Molecular genotyping and characterization of single nucleotide polymorphisms (SNPs) used in drug resistance monitoring could provide red flags of threats to continued use of SP in strategies planned by countries. This study provides data for trend profiling of molecular markers of resistance to antifolate drugs from isolates of *Plasmodium falciparum* from stored patients’ DBS obtained between 2010 and 2014 in Lagos, South-West, Nigeria.

## Methods

### Study area

**Fig. 1:**
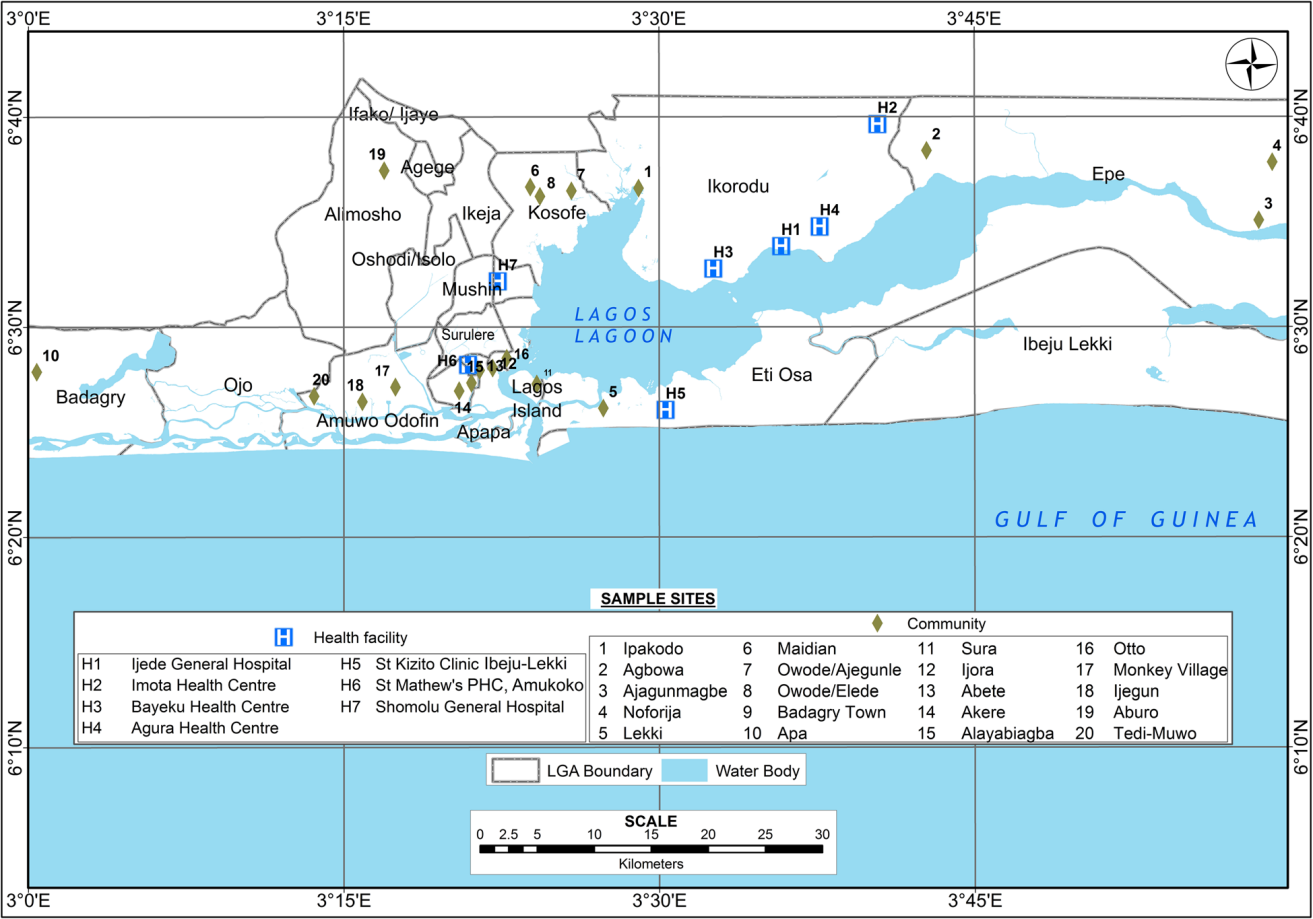
Location of health facilities and communities that were studied in Lagos State, Nigeria

The study was conducted in Lagos State, Nigeria. Lagos State is an African megacity located in south-western Nigeria on the west coast of Africa, within latitudes 6023′N and 6041′N and longitudes 2042′E and 3042′E (Fig. 1) and has an estimated population of over 10 million inhabitants, which is more than 10% of the total population of Nigeria. The state is a low-lying coastal State and Nigeria’s centre of commerce, accounting for more than 70% of the nation’s industrial and commercial establishments. Lagos is a centre of commerce with very diverse and fast-growing population, with high migration to its cities from all parts of Nigeria as well as neighbouring and foreign countries.

There are 20 Local Government Areas (LGAs) from where 50 Local Council Development Areas (LCDAs) were carved for ease of administration. The land surface generally slopes gently downwards from north to south and is naturally made up of depositional landforms which include: wetlands, barrier islands, beaches, low-lying tidal flats and estuaries [29]. Furthermore, Lagos is hypo-endemic for malaria during the dry season with moderate and stable transmission but peaks during the wet season due to increase in the population of mosquitoes [30].

### Study population and sites

The samples used for this study were obtained from children and adult patients that presented with fever/symptoms of malaria in the last 48 h in a cross-sectional case management study in health facilities (January 2013 to February 2014) and from asymptomatic persons in community surveys (October 2010 to September 2011). The health facilities were: Ijede General Hospital (H1), Imota Primary Health Centre (H2), Bayioku Primary Health Centre (H3), Agura Primary Health Centre (H4). These four health centres are situated in Ikorodu LGA. St. Kizito Primary Health Centre, Lekki in Ibeju Lekki LGA (H5), St Mathew Primary Health Centre (Catholic), Ajegunle, Amukoko in Ajeromi Ifelodu LGA (H6), and in Shomolu General Hospital, Shomolu LGA (H7) (Fig. 1). The asymptomatic study was conducted in persons aged 2 months and above in communities that were randomly and purposively selected based on malaria endemicity data, and enrolment was done using multi-stage and stratified sampling in households. The communities were: 1) Ipakodo in Ikorodu LGA, 2) Agbowa, 3) Ajagunmagbe, 4) Noforija in Epe LGA, 5) Lekki in Ibeju Lekki LGA, 6) Maidian Community, 7) Owode/Ajegunle, 8) Owode/Elede in Kosofe LGA, 9) Badagry town, 10) Apa in Badagry LGA, 11) Sura in Lagos Island LGA, 12) Ijora, 13) Abete, 14) Akere, 15) Alayabiagba in Apapa LGA, 16) Otto in Lagos Mainland LGA, 17) Monkey Village, 18) Ijegun in Amuwo Odofin LGA, 19) Aburo in Alimosho LGA, 20) Tedi-Muwo in Ojo LGA (Fig. 1).

### Preparation of dry blood spots

Dry blood spots were prepared from approximately 3–4 drops of blood collected from the study participants (both in the health facility and community) on filter paper (Whatman® filter paper #3, Whatman International Ltd., Maidstone, England). Thick and thin malaria blood films (MBFs) for malaria microscopy were also prepared for each person. The prepared blood spots were air-dried and kept in a zip-lock bag with desiccant and stored at 2–8 °C for molecular analysis.

### Malaria microscopy

Malaria microscopy on the prepared MBFs was done using standard protocol and read by independent microscopist to identify malaria positive smears [31]. The results of the malaria microscopy were used in the selection of DBS for the molecular profiling of *P. falciparum*-resistant genes and haplotypes to SP.

### DNA extraction and PCR amplification

A subset of 404 dB from the samples positive for *P. falciparum* mono infection was used for this study. Three milliliter diameter punches were made from the DBS with single-hole paper puncher. Sterilization of the puncher was done after every punch of each patient’s DBS with 70% soaked alcohol swab. The punched blood spot was placed into a 1.5 ml micro centrifuge tube. The genomic DNA from the DBSs was extracted using the QIAamp® DNA Mini kit (Qiagen, Germany) according to the manufacturer’s instructions. The DNA extracted was finally eluted using 50 μl elution buffer and kept at − 20 °C.

Nested PCR was used to amplify the extracted DNA. The primary and secondary amplification were done in a 25 μl reaction mixture that comprised of 2 μl of template genomic DNA, 1 μl of primer F, 1 μl of primer R, 12.5 μl of Taq 2x DNA master mix (Sangon Bio Inc., Shanghai, China) and 8.5 μl of double distilled water (ddH2O) using specific primers and cycling condition for the different genes.

The *dhfr* genes were amplified using the primer as previously described [32]; Forward: 5′-TCCTTTTTATGATGGAACAAG-3′, Reverse: 5′-AGTATATACATCGCTAACAGA-3′, and cycling conditions; initial denaturation for 5 min at 94 °C; 35 cycles at 95 °C for 30 s, 50 °C for 30 s and 68 °C for 1 min, finial extension at 68 °C for 5 min. 2 μl of the PCR product was used in the second round amplification with specified primers; Forward: 5′-TTTATGATGGAACAAGTCTGC-3′, Reverse: 5′-ACTCATTTTCATTTATTTCTGG-3′, and cycling conditions; 5 min at 94 °C, 30 cycles at 94 °C for 30 s, 52 °C for 30 s and 68 °C for 1 min, finial extension at 68 °C for 5 min.

The *dhps* genes were amplified using the primer as previously described [33]; Forward: 5′-AACCTAAACGTGCTGCTGTTCAA-3′, Reverse: 5′-AATTGTGTGATTTGTCCACAA-3′, and cycling conditions; initial denaturation for 5 min at 95 °C, 35 cycles at 95 °C for 30 s, 50 °C for 30 s and 68 °C for 1 min, finial extension at 68 °C for 5 min. 2 μl of the PCR product was used in the second round amplification with specified primers; Forward: 5′-ATGATAAATGAAGGTGCTAG-3′, Reverse: 5′-TCATTTTGTTGTTCATCATGT-3′, and cycling conditions; 5 min at 95 °C, 30 cycles at 95 °C for 30 s, 52 °C for 30 s and 68 °C for 1 min, finial extension at 68 °C for 5 min. The expected PCR product is 647 bp.

Positive controls were obtained from BEI Resources, USA (parasite Genomic DNA IPC 3663/3D7 strains and Dd2_R539T/Dd2 strains) and were used as references for susceptible and resistant genotypes, respectively. Nuclease-free water was used as a negative control.

The nested PCR products for *dhfr* and *dhps* were loaded on a 1% agarose gel containing 0.5 μg/ml ethidium bromide. Amplified bands of DNA were visualized under ultra-violet illumination and positive samples were selected for sequencing. The amplicon for the different genes were sequenced on an Applied Biosystem 3130 x l Genetic analyzer (Applied Biosystems, Foster City, CA, USA). Sequence alignment was done with DNASTAR 7.1 software and analyzed using the reference: *Plasmodium falciparum* 3D7 *dhps* sensitive strain (NCBI reference Sequence: XM_001349382.1) and *dhfr* sensitive strain (NCBI Reference Sequence: XM_001351443.1), respectively.

### Ethical considerations

The participants (in the health facility and community) gave written consent to participate and for their blood samples to be used for further malaria testing. Participants who presented at the screening for malaria that did not agree to participate were also attended to and standard care provided appropriately. All samples had only study identification numbers that could not be linked with personal details of the participants. The study protocol was approved by the Research Grants and Experimentation Ethics Committee, College of Medicine, University of Lagos, Nigeria and the Ethics Committee of the National Institute of Parasitic Diseases (NIPD), China.

## Results

### Demographic characteristics

Point mutations in *dhps* and *dhfr* were evaluated in 242 and 167 out of a cohort of 338 malaria-positive DBS obtained from symptomatic and asymptomatic individuals respectively from the study areas in Lagos, South – West, Nigeria between 2010 and 2014. The age of the participants were two months and above (range: 2 months–65 years; mean ± SD, 16.8 ± 14.1 years) (Table 1). The asexual parasitaemia of the study individuals ranged from 63 to 202 010 parasites/μl of blood (geometric mean parasite density of 7615 parasites/μl of blood).

**Table 1:** Demographic characteristics of the cohort of individuals with positive dry blood spots that were used in the study in Lagos, Nigeria

Description	*n* = 338
Sex, *n* (%)	
Male	152 (45.0)
Female	186 (55.0)
Age category, *n* (%)	
≤ 5 years	68 (20.1)
6–10 years	116 (34.3)
> 10 years	154 (45.6)
Parasite density (parasites/μl of blood)	
Range	63–202 010
Geometric mean	7615

### Prevalence of individual point mutations in *dhfr* and *dhps*

The sequence analyses of *dhfr* at each codon showed that the 51I mutations appeared in 95.5% of the *P. falciparum* isolates. The prevalence of 59R and 108 N mutations was 96.3% and 96.7% respectively. However, the prevalence of *P. falciparum* isolates with wild *dhfr* was low and ranged from 3.3% (108 N) to 4.5% (51I) (Table 2).

**Table 2:** Prevalence of *dhfr* and *dhps* SNPs among *Plasmodium falciparum* isolates from Lagos, Nigeria

Gene	SNPs	Wild type *n* (%)	Mutation *n* (%)
*dhfr* (*n* = 242)	51	11 (4.5)	231 (95.5)
	59	9 (3.7)	233 (96.3)
	108	8 (3.3)	234 (96.7)
	83	241 (99.6)	1 (0.4)
	122	241 (99.6)	1 (0.4)
	160	241 (99.6)	1 (0.4)
*dhps* (*n* = 167)	431	125 (74.9)	42 (25.1)
	436	95 (56.9)	69 (41.3)
	437	7 (4.2)	160 (95.8)
	540	165 (98.8)	2 (1.2)
	581	115 (68.9)	52 (31.1)
	613	97 (58.1)	70 (41.9)

*SNPs* Single nucleotide polymorphisms interval.

Furthermore, the sequence analyses of *dhps* showed that the most prevalent mutation of the cohort of individuals examined was 437G (95.8%). Other mutations were: A613S (41.9%), S436A (41.3%), A581G (34.1%), I431V (25.1%) and K540E (1.2%) (Table 2). The genetic data from this study is deposited at the National Center for Biotechnology Information (NCBI). Accession numbers- BankIt2291211: MN985140–MN985306 (167 sequences) and BankIt2298803: MT140637–MT140882 (241 Sequences).

The frequency of *dhfr* and *dhps* mutations in the health facilities and communities occurred in varied proportions (Tables 3, 4, 5, 6). The health facility situated in Ijede, Ikorodu LGA had the highest number of samples and as well as the highest frequency of *dhfr* gene mutation at codons 51I, 59R and 108 N (Table 3) and *dhps* gene mutation at codons 431 V, 436A, 437G, 581G and 613S (Table 4). Analysis of *dhfr* gene mutation among *P falciparum* isolates from asymptomatic individuals from different communities showed that Lekki located in Ibeju-Lekki LGA had the highest frequency of mutations at codons 51I, 59R and 108 N (Table 5) and *dhps* mutations at codons 431, 436, 437, 540, 581 and 613 (Table 6). Generally, there was no considerable difference in the occurrence of *dhfr* and *dhps* mutation across the sites in Lagos State.

**Table 3:** Distribution of *dhfr* mutations at the health facilities in Lagos, Nigeria

LGA/ Health facility	Location of the health facilities	No. of samples sequenced for *dhfr* *n* (%)	*dhfr* mutation *n* (%)			
			Wild	N51I	C59R	S108N
Ikorudu	Ijede [H1]	72 (34.0)	3 (1.4)	69 (32.5)	69 (32.5)	69 (32.5)
	Imota [H2]	27 (12.7)	1 (0.5)	26 (12.3)	26 (12.3)	26 (12.3)
	Bayioku [H3]	7 (3.3)	0	7 (3.3)	7 (3.3)	7 (3.3)
	Agura [H4]	52 (24.5)	3 (1.4)	49 (23.1)	48 (22.6)	49 (23.1)
Ajeromi -Ifelodun	Amukoko [H6]	35 (16.5)	0	35 (16.5)	33(15.6)	35 (16.5)
Shomolu	Shomolu [H7]	19 (9.0)	0	19 (9.0)	19 (9.0)	19 (9.0)
Total		212 (100)	7 (3.3)	205(96.7)	202(95.3)	205(96.7)

*LGA* Local Government Area.

**Table 4:** Distribution of *dhps* mutations at the health facilitie in Lagos, Nigeria

LGA/ Health facility	Location of health facilities	No. of samples sequenced for *dhps* *n* (%)	*dhps* mutation *n* (%)					
			I431V	A436S	A437G	K540E	A581G	A613S
Ikorudu	Ijede [H1]	50 (34.0)	12 (8.2)	28 (19.0)	45 (30.6)	0	14 (9.5)	20 (13.6)
	Imota [H2]	17 (11.6)	5 (3.4)	9 (6.1)	16 (10.9)	0	5 (3.4)	8 (5.4)
	Bayioku [H3]	5 (3.4)	0	4 (2.7)	5 (3.4)	0	1 (0.7)	1 (0.7)
	Agura [H4]	26 (17.7)	5 (3.4)	16 (10.9)	24 (16.3)	0	7 (4.8)	13 (8.8)
Ajeromi- Ifelodun	Amukoko [H6]	31 (21.1)	9 (6.1)	15 (10.2)	28 (19.0)	0	11 (7.5)	15 (10.2)
Shomolu	Shomolu [H7]	18 (12.2)	2 (1.4)	14 (9.5)	18 (12.2)	2 (1.4)	4 (2.7)	5 (3.4)
Total		147 (100)	33 (22.4)	83 (56.5)	136 (92.5)	2 (1.4)	42 (28.6)	62 (42.2)

*LGA* Local Government Area.

**Table 5:** Distribution of *dhfr* mutations at the community locations in Lagos, Nigeria

LGA/ Community	Name of community	No. of samples sequenced for *dhfr* *n* (%)	*dhfr* mutation *n* (%)			
			Wild	N51I	C59R	S108N
Ikorodu	Ipakodo [1]	2 (6.7)	0	2 (6.7)	2 (6.7)	2 (6.7)
Epe	Agbowa [2]	5 (16.7)	0	5 (16.7)	5 (16.7)	5 (16.7)
	Noforija [4]	1 (3.3)	0	1 (3.3)	1 (3.3)	1 (3.3)
Ibeju Lekki	Lekki [5]	10 (33.3)	1 (3.3)	9 (30.0)	9 (30.0)	9 (30.0)
Kosofe	Madian Community [6]	1 (3.3)	0	1 (3.3)	1 (3.3)	1 (3.3)
	Owode/Ajegunle [7]	3 (10.0)	0	3 (10.0)	3 (10.0)	3 (10.0)
	Owode/Elede [8]	1 (3.3)	0	1 (3.3)	1 (3.3)	1 (3.3)
Lagos Island	Sura [11]	1 (3.3)	0	1 (3.3)	1 (3.3)	1 (3.3)
Apapa	Ijora [12]	1 (3.3)	0	1 (3.3)	1 (3.3)	1 (3.3)
	Akere [14]	1(3.3)	0	1 (3.3)	1 (3.3)	1 (3.3)
Amowo Odofin	Monkey Village [17]		0	1 (3.3)	1 (3.3)	1 (3.3)
	Ijegun [18]		0	3 (10.0)	3 (10.0)	3 (10.0)
	Total	30 (100)	1 (3.3)	29 (96.7)	29 (96.7)	29 (96.7)

*LGA* Local Government Area.

**Table 6:** Distribution of *dhps* mutations at the community locations in Lagos, Nigeria

LGA/ Community	Name of community	No. of samples sequenced for *dhps* *n* (%)	*dhps* mutation *n* (%)					
			I431V	A436S	A437G	K540E	A581G	A613S
Ikorudu	Ipakodo [1]	1 (5.0)	0	0	1 (5.0)	0	0	1 (5.0)
Epe	Agbowa [2]	3 (15.0)	2 (10.0)	1 (5.0)	3 (15.0)	0	2 (10.0)	2 (10.0)
Ibeju- Lekki	Lekki [5]	9 (45.0)	2 (10.0)	4 (20.0)	9 (45.0)	0	2 (10.0)	5 (25.0)
Kosofe	Owode/Ajegunle [7]	1 (5.0)	0	1 (5.0)	1 (5.0)	0	0	0
	Owode /Elede [8]	1 (5.0)	0	1 (5.0)	1 (5.0)	0	0	0
Apapa	Akere [14]	1 (5.0)	0	1 (5.0)	1 (5.0)	0	0	1 (5.0)
	Alayabiagba [15]	1 (5.0)	0	1 (5.0)	1 (5.0)	0	0	0
Amuwo- Odofin	Monkey village [17]	2 (10.0)	0	2 (10.0)	2 (10.0)	0	0	0
	Ijegun [18]	1 (5.0)	0	1 (5.0)	1 (5.0)	0	0	0
Total		20 (100)	4 (20.0)	11(55.0)	20 (100.0)	0	4 (20.0)	9 (45.0)

*LGA* Local Government Area.

### Prevalence of *dhfr* and *dhps* haplotypes

Seven and seventeen distinct haplotypes occurred in *dhfr* and *dhps*, respectively in the cohorts of individuals in the study areas in Lagos (Table 7).

**Table 7:** Prevalence of *dhfr* and *dhps* haplotypes in *Plasmodium falciparum* isolates from Lagos, Nigeria

Gene	Category	Haplotype	*n* (%)
*dhfr*	Wild type	CNCSI	8 (3.3)
	Double mutation	CICNI	1 (0.4)
		CNRNI	3 (1.2)
	Triple mutation	CIRNI	227 (93.8)
	Quadruple mutation	CIRNIR	1 (0.4)
		CIRNIK	1 (0.4)
		CIRNIE	1 (0.4)
*dhps*	Single mutation	SGKAA	74 (44.3)
		AAKAA	4 (2.4)
	Double mutations	AGKAA	7 (4.2)
		FAKAS	3 (1.8)
		SGKGA	2 (1.2)
		AAKGA	1 (0.6)
		SGKAS	6 (3.6)
		SGEAA	2 (1.2)
		VSGKAA	1 (0.6)
	Triple mutations	SGKGS	9 (5.4)
		VAGKAA	1 (0.6)
		AGKGA	1 (0.6)
		AGKAS	14 (8.4)
	Quadruple	AGKGS	2 (1.2)
		VAGKAS	3 (1.8)
		VAGKGA	4 (2.4)
	Quintuple	VAGKGS	33 (19.8)

Mutations in the haplotype are underlined.

#### Dhfr haplotypes

Triple mutations in the *dhfr* haplotype (CIRNI) was the most prevalent (93.8%, 227/242) while 3.3% (8/242) of the isolates were wild type haplotype (CNCSI). The prevalence of double mutations in CICNI and CNRNI was 0.4% (1/242) and 1.2% (3/242) respectively while 1.2% of the samples had quadruple mutations at positions: 83R, 122 K and 160E together with the triple CIRNI mutations (Table 7).

#### Dhps haplotypes

Mutations in the *dhps* haplotype occurred in different proportions: single mutation in SGKAA was 44.3% (74/167). Quintuple mutations in VAGKGS (19.8%, 33/167) was the most prevalent of the multiple mutations compared to the triple, double and quadruple mutations that were recorded (Table 7).

#### *Dhfr* and *dhps* allele combinations

A total of 19 haplotypes were seen in the *dhfr* and *dhps* combinations (Table 8). Quadruple mutations occurred in the combined *dhfr* (triple mutations) + *dhps* (single mutation) haplotype (CIRNI + SGKAA) and was the most prevalent (42.6%, 60/141). This was followed by octuple mutations in CIRNI + VAGKGS (22.0%, 31/141). Sextuple mutations (CIRNI + AGKAS) and quintuple mutations (CIRNI + AGKAA) among the isolates were 9.9% and 5.0% respectively. Mutations in the haplotypes of the other combined genotypes were generally low (Table 8).

**Table 8:** Prevalence of combined *dhfr* and *dhps* haplotypes combinations in *Plasmodium falciparum* isolates from Lagos, Nigeria

Gene	Category	Haplotype	*n* (%)
*dhfr*/*dhps* (*n* = 141)	Triple mutant	CNRNI + SGKAA	2 (1.4)
		CNRNI + AAKAA	1 (0.7)
	Quadruple Mutant	CIRNI + SGKAA	60 (42.6)
		CIRNI + AAKAA	2 (1.4)
	Quintuple mutant	CIRNI + AGKAA	7 (5.0)
		CIRNI + SGKAS	5 (3.5)
		CIRNI + SGKGA	2 (1.4)
		CIRNI + FAKAS	2 (1.4)
		CIRNI + VSGKAA	1 (0.7)
	Sextuple mutant	CIRNI + AGKAS	14 (9.9)
		CIRNI + SGKGS	4 (2.8)
		CIRNI + VAGKAA	1 (0.7)
		CIRNI + AGKGA	1 (0.7)
		CIRNI + AGKGA	1 (0.7)
		CIRNI + AGKGA	2 (1.4)
	Septuple mutant	CIRNI + VAGKGA	4 (2.8)
		CIRNI + VAGKAS	2 (1.4)
		CIRNI + AGKGS	2 (1.4)
	Octuple mutant	CIRNI + VAGKGS	31 (22.0)

Mutated alleles are underlined.

## Discussion

*P. falciparum* drug resistance remains a challenge to effective malaria case management and prevention. This has made continuous monitoring of molecular markers of antimalarial drug resistance imperative in malaria-endemic countries to track trends and distribution of relevant resistant genes and haplotypes to ensure that threats to existing artemisinin combination therapies and drug-dependent interventions are identified and addressed promptly. Information on these threats will also further guide National Malaria Control Programmes to adopt the most suitable interventions using the appropriate drug combinations. Our study showed high frequencies of *P. falciparum* isolates with mutant *dhfr* and *dhps* in circulation in Lagos, Nigeria.

The *dhfr* triple haplotype mutation (CIRNI) was highly prevalent at all study sites in our study, which was comparable with some other reported studies among pregnant women in Nigeria and in Sub Saharan Africa [9, 10, 34]. These reported studies indicating the authors, the mutation prevalance and the time of sample collection in Nigeria included: Agomo et al., 66.7% in Lagos in 2008/2009 [8], Iwalokun et al., 50.0% (Lagos) (2011) [9], and Oguike et al., 100.0% (Ibadan) (2003), 81.3% (Maiduguri) (2010), 90.2% (Enugu) (2010) and 98.7% in Benin city (2014/2015) [10]; and in Guinea, Jiang et al., 86.8% (Bioko Island) (2013/2014) [34]. There was an increase in *dhfr* triple haplotype mutations in Lagos within about 2 years. This mutation is associated with high-level resistance to pyrimethamine [35–37] and increased risk of SP resistance if it occurreds concurrently with *dhps* mutations [26, 27, 36, 37]. Mutations such as 16 V+ 164 L in dhfr that are associated with high resistance to cycloguanil, the active form of proguanil [37, 38] were not observed in our study in Lagos, Nigeria.

Mutations in *dhps* haplotypes at 437 occurred in 95.8%, 31.1% and 1.2% at codons 581 and 540 respectively in the samples analyzed. Amino acid changes at position 437 (A437G) represented the critical mutation for sulphadoxine resistance. Additional mutation(s) at positions 436 (S436A/F), 540 (K540E), 581 (A581G), and 613 (A613S/T) are associated with decreased parasite sensitivity to the sulpha drugs including sulphadoxine and dapsone [13, 18, 19]. Two (1.2%) *dhps* double haplotype mutation consisting of A437G and K540E were seen and have been consistently associated with in vivo clinical failure independently [26, 38]. Similarly, 581G *dhps* haplotype mutation has also been shown to be associated with important modulatory role in resistance [39] .The World Health Organization (WHO) recommends that when the frequency of this mutation is above 10.0%, IPTp with SP may not be able to protect pregnant women from delivering infants with low birth weight [40]. The 540E and 581G haplotype mutations have also been shown to have important implications for the effectiveness of SP in children less than 5 years of age and in pregnant women [41]. Reports from previous studies within Nigeria and in Africa were: 37.5% and 22.5% of A437G and K540E haplotype mutations respectively in Lagos (2011) [9] and 96.4% of 437G haplotype and no mutation at K540 codon in Calabar (2013/2014) [42], Nigeria. In Mukono District (Uganda), high frequency of mutation in *dhps* codon 437G (99.1%) and 540E (98.2%) (2010–2012) [43] was reported within the same period in which the samples in our study were collected.

The prevalence of 581G *dhps* haplotype mutation was (31.4%) and is associated with important modulatory role in resistance [39]. The World Health Organization (WHO) recommends that when the frequency of the 540 *dhps* haplotype mutation exceeds 95%, IPTp should not be implemented, because it could fail [40]. WHO also recommends that when the frequency of *dhps* Ala581Gly haplotype is above 10.0%, IPTp with SP may not be able to protect pregnant women from delivering infants with low birth weight [40]. Our study was in the general population and retrospectively, there were no existing data on birth outcomes to correlate this finding in the study areas where the samples were collected between 2010 and 2014. Nevertheless, it underscored the need for regular molecular marker studies in areas where SP is used for malaria interventions. In addition, It is worth noting that the occurrence of the *dhps* 540E and Ala581Gly haplotype mutations are rare in West Africa, but common in east and southern Africa [39, 44].

The I431V occurred in combination with other *dhps* haplotype mutations, and the most frequent was VAGKGS haplotype. There were similar reports on this emerging mutation on *dhps* in Nigeria and Cameroun [10, 21, 22]. The occurrence of I431V mutation in *dhps* over the years may suggest conferment of selective advantage in the presence of SP drug pressure and displacement of the more sensitive haplotypes. Since SP is used as IPTp, and also readily available in the Nigerian market for treatment of malaria [45], an indication that ongoing SP drug pressure is strong. Another plausible explanation is that *dhps* haplotype mutation (431 V) has arisen by chance and provided an improvement in the fitness of parasites carrying the 437, 581 and/or 613 mutations, but does not change susceptibility to sulphadoxine [10]. Further studies are needed to assess the effect of this mutation on the phenotype of parasites carrying this haplotype.

Partial resistance to SP [28] in *dhfr-dhps* haplotypes combinations were described in our study. The prevalence was 42.6% and 22.0% for quadruple haplotype mutations, CIRNI-SGKAA and octuple haplotype (CIRNI + VAGKGS) respectively. Specifically, CIRNI-SGKAA was highly associated with sub-optimal IPTp-SP effectiveness in previous studies [46]. However, K540E haplotype mutation was not found in any of the *dhfr-dhps* combinations in our study.

In West Africa wild *dhps* K540 commonly occur with triple *dhfr* mutations and single 437G *dhps* mutation [47, 48]. The highest levels and spread of antifolate resistance are found in Southeast Asia and South America [48–50]. In these two regions, a polymorphism at *dhfr* residue 164 is almost always found, but is rarely seen sub-Saharan Africa despite extensive use of the drug [48]. Moderate level of resistance conferred by *dhfr* and *dhps* polymorphisms is typically found in West Africa with the absence of I164L polymorphism that is associated with very high-level SP resistance (up to 20 000-fold decrease in susceptibility in comparison with the wild type) [50]. I164L polymorphism have been variously reported in parts of East Africa [51], some parts of South Africa [52] and Asia [53]. There is dearth of information on why I164L mutation does not occur in Africa despite extensive drug pressure. It was suggested that this amino acid change carries a high fitness cost to the parasite, such that it is unable to survive the immune response of “*malaria-experienced*” hosts in West Africa [50]. Nevertheless, though SP is ineffective in treating symptomatic disease in malaria-naïve children in many parts of Africa, it has retained some efficacy in preventing malaria in pregnant women [49].

For consideration that the samples we used in our study were collected nearly 10 years ago, and SP has been restricted to be used in malaria control among general population for several years in Nigeria due to severe drug resistance developed in *P. falciparum*, recovery of wild type of SP sensitive parasite could probably be expected. Similar situation has ever occurred that chloroquine sensitivity of *P. falciparum* reappeared after long time stopping of drug use in malaria control [54]. Recent investigation of SP sensitivity of malaria parasite in local area in Nigeria should be proceeded.

## Conclusion

This study showed a high prevalence of *dhfr* and *dhps* mutant alleles in *Plasmodium falciparum* isolates in Lagos, Nigeria, indicating that SP resistant parasites were in circulation five to 8 years after the introduction of ACT regimen. There was increased prevalence in *dhfr* triple haplotype mutations when compared with previous reports in the same environment but aligned with high prevalence in other locations in Nigeria and other countries in Africa. Mutation in *dhps*, particularly 540E that is scarcely reported was low in this study. Partiallyt *dhfr-dhps* haplotype mutations were reported while I164L mutation that is consistently associated with SP resistance was not seen. This study has added to the repertoire of SP haplotype research for analyses of trends and monitoring of threats to continued use of SP. Molecular marker studies on resistant genotypes and haplotypes of SP remains invaluable where the medicine is used in various interventions by national malaria programmes.

## Data Availability

Genetic data from this study deposited at the National Center for Biotechnology Information (NCBI). Accession numbers- BankIt2291211: MN985140 - MN985306 (167 sequences) and BankIt2298803: MT140637 - MT140882 (241 Sequences).

## Abbreviations

SP: Sulphadoxine–pyrimethamine
ACT: Artemisinin-based combination therapy
*Pfdhfr*: *Plasmodium falciparum* dihydrofolate reductase
*dhps*: dihydropteroate synthase
IPT: Intermittent preventive treatment
MiP: Malaria in pregnancy
SNPs: Single nucleotide polymorphism

## References

[1] WHO. Global Report on Antimalarial Drug Efficacy and Drug Resistance: 2000–2010. 2010.

[2] FMOH: Federal ministry of health N, PP. le27. National Malaria Control Programme in Nigeria. Annual Report 2005.

[3] Uhlemann AC, Krishna S (2005). Antimalarial multi-drug resistance in Asia: mechanisms and assessment. Curr Top Microbiol Immunol.

[4] Wellems TE, Plowe CV (2001). Chloroquine-resistant malaria. J Infect Dis.

[5] Wongsrichanalai C, Pickard AL, Wernsdorfer WH, Meshnick SR (2002). Epidemiology of drug-resistant malaria. Lancet Infect Dis.

[6] WHO. World Health Malaria Report. World Health Organization. 2014.

[7] Desai M, Gutman J, Taylor SM, Wiegand RE, Khairallah C, Kayentao K (2016). Impact of sulfadoxine-pyrimethamine resistance on effectiveness of intermittent preventive therapy for malaria in pregnancy at clearing infections and preventing low birth weight. Clin Infect Dis.

[8] Agomo CO, Oyibo WA, Sutherland C, Hallet R, Oguike M (2016). Assessment of markers of antimalarial drug resistance in *Plasmodium falciparum* isolates from pregnant women in Lagos, Nigeria. PLoS One.

[9] Iwalokun BA, Iwalokun SO, Adebodun V, Balogun M (2015). Carriage of mutant dihydrofolate reductase and dihydropteroate synthase genes among *Plasmodium falciparum* isolates recovered from pregnant women with asymptomatic infection in Lagos, Nigeria. Med Princ Pract.

[10] Oguike MC, Falade CO, Shu E, Enato IG, Watila I, Baba ES, et al. Molecular determinants of sulfadoxine-pyrimethamine resistance in *Plasmodium falciparum* in Nigeria and the regional emergence of *dhps* 431V. Int J Parasitol Drugs Drug Resist. 2016;6(3):220–9. 10.1016/j.ijpddr.2016.08.004 PMC5094156 27821281

[11] Wang P, Lee CS, Bayoumi R, Djimde A, Doumbo O, Swedberg G, et al. Resistance to antifolates in *Plasmodium falciparum* monitored by sequence analysis of dihydropteroate synthetase and dihydrofolate reductase alleles in a large number of field samples of diverse origins. Mol Biochem Parasitol. 1997;89(2):161–77. 10.1016/s0166-6851(97)00114-x 9364963

[12] Dieckmann A, Jung A (1986). Mechanisms of sulfadoxine resistance in *Plasmodium falciparum*. Mol Biochem Parasitol.

[13] Triglia T, Menting JG, Wilson C, Cowman AF. Mutations in dihydropteroate synthase are responsible for sulfone and sulfonamide resistance in *Plasmodium falciparum*. Proc Natl Acad Sci U S A. 1997;94(25):13944–9. 10.1073/pnas.94.25.13944 PMC28412 9391132

[14] Chulay JD, Watkins WM, Sixsmith DG (1984). Synergistic antimalarial activity of pyrimethamine and sulfadoxine against *Plasmodium falciparum* in vitro. Am J Trop Med Hyg..

[15] Cowman AF, Morry MJ, Biggs BA, Cross GA, Foote SJ (1988). Amino acid changes linked to pyrimethamine resistance in the dihydrofolate reductase-thymidylate synthase gene of *Plasmodium falciparum*. Proc Natl Acad Sci U S A.

[16] Contreras CE, Cortese JF, Caraballo A, Plowe CV (2002). Genetics of drug-resistant *Plasmodium falciparum* malaria in the Venezuelan state of bolivar. Am J Trop Med Hyg.

[17] Khalil I, Rønn AM, Alifrangis M, Gabar HA, Satti GM, Bygbjerg IC (2003). Dihydrofolate reductase and dihydropteroate synthase genotypes associated with in vitro resistance of *Plasmodium falciparum* to pyrimethamine, trimethoprim, sulfadoxine, and sulfamethoxazole. Am J Trop Med Hyg..

[18] Triglia T, Wang P, Sims PF, Hyde JE, Cowman AF (1998). Allelic exchange at the endogenous genomic locus in *Plasmodium falciparum* proves the role of dihydropteroate synthase in sulfadoxine-resistant malaria. EMBO J.

[19] Berglez J, Iliades P, Sirawaraporn W, Coloe P, Macreadie I. Analysis in *Escherichia coli* of *Plasmodium falciparum* dihydropteroate synthase (DHPS) alleles implicated in resistance to sulfadoxine. Int J Parasitol. 2004;34(1):95–100. 10.1016/j.ijpara.2003.09.009 14711594

[20] Pearce RJ, Pota H, Evehe MS, Bâ EH, Mombo-Ngoma G, Malisa AL, Ord R, et al. Multiple origins and regional dispersal of resistant *dhps* in African Plasmodium falciparum malaria. PLoS Med. 2009;6(4):e1000055. 10.1371/journal.pmed.1000055 PMC2661256 19365539

[21] Sutherland CJ, Fifer H, Pearce RJ, Reza F, Nicholas M, Haustein T, Njimgye-Tekumafor NE, et al. Novel *pfdhps* haplotypes among imported cases of *Plasmodium falciparum* malaria in the United Kingdom. Antimicrob Agents Chemother. 2009;53(8):3405–10. 10.1128/AAC.00024-09 PMC2715629 19433569

[22] Chauvin P, Menard S, Iriart X, Nsango SE, Tchioffo MT, Abate L, et al. Prevalence of Plasmodium falciparum parasites resistant to sulfadoxine/pyrimethamine in pregnant women in Yaoundé, Cameroon: emergence of highly resistant *pfdhfr/**pfdhps* alleles. J Antimicrob Chemother. 2015;70(9):2566–71. 10.1093/jac/dkv160 26080363

[23] Plowe CV (2009). The evolution of drug-resistant malaria. Trans R Soc Trop Med Hyg.

[24] Kun JF, Lehman LG, Lell B, Schmidt-Ott R, Kremsner PG (1999). Low-dose treatment with sulfadoxine-pyrimethamine combinations selects for drug-resistant *Plasmodium falciparum* strains. Antimicrob Agents Chemother.

[25] Kublin JG, Dzinjalamala FK, Kamwendo DD, Malkin EM, Cortese JF, Martino LM, Mukadam RAG (2002). Molecular markers for failure of sulfadoxine-pyrimethamine and chlorproguanil-dapsone treatment of *Plasmodium falciparum* malaria. J Infect Dis.

[26] Happi CT, Gbotosho GO, Folarin OA, Akinboye DO, Yusuf BO, Ebong OO, et al. Polymorphisms in *Plasmodium falciparum **dhfr* and *dhps* genes and age related in vivo sulfadoxine-pyrimethamine resistance in malaria-infected patients from Nigeria. Acta Trop. 2005;95(3):183–93. 10.1016/j.actatropica.2005.06.015 16023986

[27] Afonso A, Hunt P, Cheesman S, Alves AC, Cunha CV. Do Rosário V, Cravo P, et al. Malaria parasites can develop stable resistance to artemisinin but lack mutations in candidate genes *atp6* (encoding the sarcoplasmic and endoplasmic reticulum Ca2+ ATPase), *tctp*, *mdr1*, and *cg10*. Antimicrob Agents Chemother. 2006;50(2):480–9. 10.1128/AAC.50.2.480-489.2006 PMC1366921 16436700

[28] Naidoo I, Roper C (2013). Mapping 'partially resistant', 'fully resistant', and 'super resistant' malaria. Trends Parasitol.

[29] Odusanya OO, Akinyinka MR, Oluwole EO, Odugbemi BA, Bakare OQ, Adeniran A. How does the public perceive healthcare workers in Lagos? A comparison of health workers in public and private health facilities. Niger Postgrad Med J. 2018;25(3):177–85. 10.4103/npmj.npmj_102_18 30264770

[30] National Population Commision (NPC) (Nigeria) NMCPNNaII. Nigeria Malaria Indicator Survey 2010. Abuja, Nigeria and ICF International.

[31] WHO (1991). Basic Laboratory methods in Medical Parasitology.

[32] Alam MT, de Souza DK, Vinayak S, Griffing SM, Poe AC, Duah NO (2011). Selective sweeps and genetic lineages of *Plasmodium falciparum* drug -resistant alleles in Ghana. J Infect Diss.

[33] Vinayak S, Alam MT, Mixson-Hayden T, McCollum AM, Sem R, Shah NK (2010). Origin and evolution of sulfadoxine resistant *Plasmodium falciparum*. PLoS Pathog.

[34] Jiang T, Chen J, Fu H, Wu K, Yao Y, Eyi JUM, et al. High prevalence of *Pfdhfr*-*Pfdhps* quadruple mutations associated with sulfadoxine-pyrimethamine resistance in *Plasmodium falciparum* isolates from Bioko Island, Equatorial Guinea. Malar J. 2019;18(1):101. 10.1186/s12936-019-2734-x PMC6434785 30914041

[35] Basco LK, Eldin de Pécoulas P, Wilson CM, Le Bras J, Mazabraud A. Point mutations in the dihydrofolate reductase-thymidylate synthase gene and pyrimethamine and cycloguanil resistance in *Plasmodium falciparum*. Mol Biochem Parasitol. 1995;69(1):135–8. 10.1016/0166-6851(94)00207-4 7723784

[36] Nzila-Mounda A, Mberu EK, Sibley CH, Plowe CV, Winstanley PA, Watkins WM (1998). Kenyan *Plasmodium falciparum* field isolates: correlation between pyrimethamine and chlorcycloguanil activity in vitro and point mutations in the dihydrofolate reductase domain. Antimicrob Agents Chemother.

[37] Foote SJ, Kyle DE, Martin RK, Oduola AM, Forsyth K, Kemp DJ, Cowman AF (1990). Several alleles of the multidrug-resistance gene are closely linked to chloroquine resistance in *Plasmodium falciparum*. Nature.

[38] Peterson DS, Milhous WK, Wellems TE (1990). Molecular basis of differential resistance to cycloguanil and pyrimethamine in *Plasmodium falciparum* malaria. Proc Natl Acad Sci U S A.

[39] Gutman J, Kalilani L, Taylor S, Zhou Z, Wiegand RE, Thwai KL (2015). The A581G mutation in the gene encoding *Plasmodium falciparum* dihydropteroate synthetase reduces the effectiveness of sulfadoxine-pyrimethamine preventive therapy in Malawian pregnant women. J Infect Dis.

[40] WHO. WHO Evidence Review Group on Intermittent Preventive Treatment (IPT) of malaria in pregnancy. Geneva: World Health Organization; 2013.

[41] Chico RM, Cano J, Ariti C, Collier TJ, Chandramohan D, Roper C (2015). Influence of malaria transmission intensity and the 581G mutation on the efficacy of intermittent preventive treatment in pregnancy: systematic review and meta-analysis. Tropical Med Int Health.

[42] Esu E, Tacoli C, Gai P, Berens-Riha N, Pritsch M, Loescher T, et al. Prevalence of the *Pfdhfr* and *Pfdhps* mutations among asymptomatic pregnant women in Southeast Nigeria. Parasitol Res. 2018;117(3):801–7. 10.1007/s00436-018-5754-5 29332155

[43] Mbonye AK, Birungi J, Yanow SK, Shokoples S, Malamba S, Alifrangis M (2015). Prevalence of *Plasmodium falciparum* resistance markers to sulfadoxine-pyrimethamine among pregnant women receiving intermittent preventive treatment for malaria in Uganda. Antimicrob Agents Chemother.

[44] van Eijk AM, Larsen DA, Kayentao K, Koshy G, Slaughter DEC, Roper C (2019). Effect of *Plasmodium falciparum* sulfadoxine-pyrimethamine resistance on the effectiveness of intermittent preventive therapy for malaria in pregnancy in Africa: a systematic review and meta-analysis. Lancet Infect Dis.

[45] Ugwu EO, Iferikigwe ES, Obi SN, Ugwu AO, Agu PU, Okezie OA (2013). Anti-malaria prescription in pregnancy among general practitioners in Enugu state, south East Nigeria. Niger Med J.

[46] Minja DT, Schmiegelow C, Mmbando B, Boström S, Oesterholt M, Magistrado P (2013). *Plasmodium falciparum* mutant haplotype infection during pregnancy associated with reduced birthweight, Tanzania. Emerg Infect Dis.

[47] Mugittu K, Ndejembi M, Malisa A, Lemnge M, Premji Z, Mwita A (2004). Therapeutic efficacy of sulfadoxine-pyrimethamine and prevalence of resistance markers in Tanzania prior to revision of malaria treatment policy: *Plasmodium falciparum* dihydrofolate reductase and dihydropteroate synthase mutations in monitoring in vivo resistance. Am J Trop Med Hyg..

[48] Sibley CH, Hyde JE, Sims PF, Plowe CV, Kublin JG, Mberu EK (2001). Pyrimethamine-sulfadoxine resistance in *Plasmodium falciparum*: what next?. Trends Parasitol.

[49] ter Kuile FO, van Eijk AM, Filler SJ (2007). Effect of sulfadoxine-pyrimethamine resistance on the efficacy of intermittent preventive therapy for malaria control during pregnancy: a systematic review. JAMA.

[50] Nzila AM, Mberu EK, Nduati E, Ross A, Watkins WM, Sibley CH (2002). Genetic diversity of *Plasmodium falciparum* parasites from Kenya is not affected by antifolate drug selection. Int J Parasitol.

[51] Lynch CA, Pearce R, Pota H, Egwang C, Egwang T, Bhasin A (2017). Travel and the emergence of high-level drug resistance in *Plasmodium falciparum* in Southwest Uganda: results from a population-based study. Malar J.

[52] Kaingona-Daniel EP, Gomes LR, Gama BE, Almeida-de-Oliveira NK, Fortes F, Ménard D (2016). Low-grade sulfadoxine-pyrimethamine resistance in *Plasmodium falciparum* parasites from Lubango, Angolia. Malar J.

[53] Basuki S, Fitriah RPM, Kasmijati AP, Riyanto S (2018). Origins and spread of novel genetic variants of sulfadoxine-pyrimethamine resistance in *Plasmodium falciparum* isolates in Indonesia. Malar J.

[54] Egan TJ, Kaschula CH (2007). Strategies to reverse drug resistance in malaria. Curr Opin Infect Dis.

